# Prognostic value of preoperative inflammation-based predictors in patients with bladder carcinoma after radical cystectomy

**DOI:** 10.1515/med-2021-0277

**Published:** 2021-05-21

**Authors:** Huiming Gui, Yutong Song, Yongsheng Yin, Hanzhang Wang, Ronald Rodriguez, Zhiping Wang

**Affiliations:** Institute of Urology, Lanzhou University Second Hospital, Chengguan District, 82 Cuiying Gate, Lanzhou University, Lanzhou 730000, P. R. China; Department of Urology, Gansu Provincial People’s Hospital, Lanzhou, China; Department of Urology, University of Texas Health Science Center at San Antonio, 7703 Floyd Curl Drive, San Antonio, TX 78229-3900, United States of America

**Keywords:** plasma fibrinogen, neutrophil–lymphocyte ratio, inflammatory biomarker, prognosis, bladder carcinoma

## Abstract

**Aims:**

Emerging evidence has related inflammation-based biomarkers to numerous carcinomas, including bladder carcinoma (BC). However, the role of inflammatory biomarkers in the prognosis of BC remains inconclusive. This study aimed to compare preoperative plasma fibrinogen (PF) and other inflammatory biomarkers such as the platelet–lymphocyte ratio (PLR), neutrophil–lymphocyte ratio (NLR), lymphocyte–monocyte ratio (LMR), C-reactive protein (CRP) level, and serum albumin level to predict the prognosis of patients with BC.

**Methods:**

This article focused on a retrospective analysis of 175 patients with newly diagnosed BC who were admitted to our hospital from March 2005 to March 2016. Of these BC patients, 136 had undergone radical cystectomy (RC).

**Results:**

According to multivariate analysis, high PF level was an independent predictor of overall survival (OS) in 136 BC patients receiving RC (HR = 3.759; *P* = 0.011), but not for all 175 BC patients. Combining the NLR and PF values showed higher predictive accuracy for OS than NLR or PF alone (*P* < 0.05). Additionally, for 136 BC patients who had undergone RC, a close relationship was found between high PF levels (≥3.39 g/L) and lymph node metastasis (*P* = 0.011) and clinical T stage (*P* = 0.015). Furthermore, PF was a superior prognostic factor compared with the LMR, PLR, CRP, and albumin values in 136 BC patients who had undergone RC (*P* < 0.001).

**Conclusions:**

The preoperative PF level may be a prognostic biomarker; and when combined with the NLR, it can improve the predictive ability of the survival of BC patients, particularly of BC patients who underwent RC.

## 1 Introduction

Bladder carcinoma (BC) ranks 11th among all malignancies worldwide. Globally, the age-standardized incidence rates of BC (100,000 per person/year) are 9.0 for men and 2.2 for women; the age-standardized mortality rates (100,000 per person/years) are 3.2 for men versus 0.9 for women [1]. Approximately 70% of bladder tumours is confined to the mucosa (stage Ta and CIS) or submucosa (stage T1), and 30% is muscle-invasive BC ([MIBC]; T2–T4) [2]. Radical cystectomy (RC), the standard treatment of MIBC, is characterized by low mortality and an acceptable incidence of complications and is the first-choice treatment of T2–T4 BC at many institutions [3,4]. Although the survival rates of BC patients have improved in recent decades, still tumour recurrence and progression occur in many patients. The role of some prognostic factors, including the neutrophil–lymphocyte ratio (NLR), serum D-dimer, and plasma fibrinogen (PF), has been confirmed in BC [5,6]. However, thus far, most of these markers have not clinically been used because they have been ineffective in predicting the prognosis in patients. Additionally, biomarker analysis of pathologic specimens may allow for enhanced risk stratification and guide better prognostic evaluations for a more effective therapeutic strategy.

During human tumour development, inflammation plays a key role [7]. Current hypotheses suggest that the synthesis of inflammatory cytokines may be stimulated by the tumour microenvironment, resulting in increases in acute phase reactants and serum parameters involving lymphocytes and neutrophils [8]. The platelet–lymphocyte ratio (PLR), NLR, C-reactive protein (CRP) level, lymphocyte–monocyte ratio (LMR), albumin level, and PF level are emerging biomarkers of host inflammation in various malignancies [9,10,11,12]. However, few studies have simultaneously compared their prognostic value in BC patients.

Previous studies reported that many tissue- or urine-based markers can predict the prognosis of BC; most of these are based on PCR or immunohistochemical methods [13]. A promising, simple, and economical approach is ideal for future clinical assessments. The PF, NLR, LMR, PLR, CRP, and albumin values, which can easily be detected from the whole blood cell count, have been suggested as predictors and can estimate the magnitude of systemic inflammation in critically ill patients [14,15,16,17] and assess the survival outcomes of several solid malignancies [18,19,20].

As a marker of systemic inflammation, PF is often associated with haemostasis and plays a key role in tumour research [21,22]. The pretreatment PF level has been shown to predict oncologic outcomes for digestive system tumours, lung carcinoma, gynaecologic carcinoma, and glioblastomas, but studies of its prognostic value in BC are lacking [18,23,24,25]. Additionally, at this stage, the potential molecular mechanism of PF affecting tumorigenesis remains controversial. We aimed to determine the value of PF for predicting oncologic outcomes in BC patients compared with that of other inflammation-based indices (CRP and albumin) and cellular parameters (NLR, LMR, and PLR).

## 2 Patients and methods

### 2.1 Patients

The study was conducted from March 2005 to March 2016 and included 175 consecutive patients with pathologically confirmed BC and also included 136 BC patients who had undergone RC at a single academic hospital. Blood cells were counted 7 days before the surgery. The included criteria are as follows: (1) a confirmed BC histology, (2) a survival time after surgery of longer than 30 days, and (3) extensive follow-up data and medical records. Exclusion criteria included chronic inflammation, acute infection, or blood cell effects. Finally, 175 patients were included in the study. The study was approved by the Ethics Committee of the Second Hospital of Lanzhou University (Ethical Application number: 2016A-070). Each included patient was informed about the study in writing and agreed to the research agreement.

### 2.2 Clinicopathological features and follow-up

The clinicopathological records were reviewed to determine the tumour category and grade, clinical T stage, tumour diameter (<3 vs >3 cm), presence of comorbidities, history of adjuvant chemotherapy, lymphovascular invasion, radiotherapy focality, lymph node metastasis (LNM), and putative preoperative risk factors (preoperative PF, NLR, CRP, LMR, albumin, PLR, age at diagnosis, and gender). The tumour stage and grade were based on the 2004 TNM staging system and 1997 WHO classification, respectively. The follow-up data were obtained from telephone interviews, guardians, and patient files. The primary outcome of the study was overall survival (OS), which refers to the period from the time of surgery to the last visit or death from any cause. Patient follow-up was completed up to February 2021.

### 2.3 Optimal prognostic cutoff values for PF and NLR

**Figure S1:**
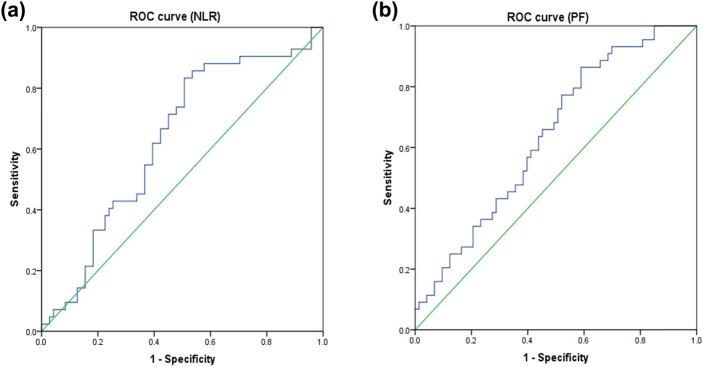
The ROC curves of NLR (a) and PF (b) for overall survival (OS) in bladder carcinoma.

We determined the optimal cutoff value using the minimum *p* value method (Galon et al., 2006). Among the prognostic scores, the optimum cutoff values were 3.39 g/L for preoperative PF and 3.05 for preoperative NLR; and the areas under ROC curve (AUCs) corresponding to PF and NLR and representing the maximum values were 0.639 and 0.630, respectively (Figure S1). The patients were divided into two groups according to the ROC threshold. The higher group exhibited values greater than the optimal cutoff levels, whereas the lower group exhibited values less than the optimal cutoff levels. Among 175 patients, the corresponding median PF value and mean PF were 3.29 and 2.96 g/L, respectively. The median NLR and mean NLR values in 175 patients were 2.86 and 2.67, respectively. One hundred seventy-five patients were divided by CRP, LMR, PLR, and albumin levels using the optimized cutoff values to predict OS, as derived from the ROC curve analysis (<3.06, >138, >5.0 mg/L, and <3.5 g/dL, respectively). The AUCs for LMR, CRP, PLR, and albumin regarding OS were 0.660 (95% CI: 0.624–0.695), 0.635 (95% CI: 0.587–0.650), 0.624 (95% CI: 0.586–0.655), and 0.652 (95% CI: 0.616–0.687), respectively.

### 2.4 Inflammatory parameters

Blood samples were collected by peripheral venous puncture between the diagnosis and initiation of treatment and were determined as follows: cell count ratios (PLR, NLR, and LMR), serum albumin levels, PF concentration, serum CRP, and individual cell counts (platelets, monocytes, neutrophils, and lymphocytes). Sample collections were usually performed 1 week before treatment initiation (median of 5 days; IQR = 2–8 days) as part of the routine pretreatment clinical assessment. The normal ranges for albumin, CRP, and fibrinogen were 0–0.5, 0–10, and 200–400 mg/dL, respectively.

### 2.5 Statistical analysis

Statistical analyses were performed using the log-rank test and Kaplan–Meier method. The association between OS and each clinicopathological variable was analysed using the multivariate Cox regression model and univariate Cox regression model. A *P* value less than 0.05 indicated a statistically significant level. Statistical analysis was performed using version 22.0 of the Statistical Package for the Social Sciences (SPSS Inc., New York, US).

## 3 Results

### 3.1 Patient characteristics

Among 175 BC patients, 136 patients had undergone RC. The proportion of male patients was much higher than that of female patients – 124 (70.9%) and 51 (29.1%), respectively. The average age of 175 patients at the time of treatment was 59.5 ± 6.7 years. The 5-year OS rate for 175 BC patients was 68.6%. Information on the treatment characteristics, clinical, and histopathology of these patients are provided in Table 1. The demographic characteristics of all 175 patients are shown in Table S1. Among 136 patients who had received RC, the demographic characteristics are shown in Table 3.

**Table 1:** Univariate and multivariate analyses of characteristics associated with OS in all 175 BC patients

Characteristics	Univariate			Multivariate		
	Hazard ratio	95% CI	*P* value	Hazard ratio	95% CI	*P* value
**Age, years**						
≥60 vs <60	1.065	0.669–1.696	0.790			
**Gender**						
Male vs Female	0.990	0.592–1.656	0.970			
**Clinical T stage**						
>T2 vs ≤T2	2.790	1.737–4.483	**<0.001**	2.320	1.368–3.935	**0.002**
**Pathological grade**						
G2–G3 vs G1	1.764	1.105–2.816	**0.017**			
**Histological subtype**						
UBC vs Non-UBC	1.682	0.807–3.506	0.166			
**LNM**						
Yes vs No	3.529	2.147–5.802	**<0.001**	1.869	1.044–3.345	**0.035**
**PF**						
≥3.39 vs <3.39 g/L	2.372	1.402–4.011	**0.001**			
**NLR**						
≥3.05 vs <3.05	2.513	1.46–0.309	**0.001**	4.419	1.615–12.088	**0.004**
**LMR**						
<3.06 vs ≥3.06	2.117	1.302–3.440	**0.002**			
**PLR**						
≥138 vs <138	2.458	1.491–4.053	**<0.001**			
**CRP**						
≥5.0 vs <5.0 mg/L	2.639	1.565–4.448	**<0.001**			
**Serum albumin**						
≥3.5 vs <3.5 g/dL	2.153	1.316–3.522	**0.002**			
**PF + NLR**						
PF high + NLR high vs PF high or NLR high vs PF low + NLR low	4.121	1.687–10.070	**0.002**	0.294	0.091–0.953	**0.041**

*P* values that achieved statistical significance (*P* < 0.05) are indicated in boldface. Abbreviations: CRP = C-reactive protein; LMR = lymphocyte–monocyte ratio; LNM = lymph node metastasis; NLR = neutrophil–lymphocyte ratio; PF = plasma fibrinogen; PLR = platelet–lymphocyte ratio; UBC = urothelial bladder carcinoma.

### 3.2 Univariate survival analysis of all prognostic parameters

We used univariate analysis to assess the risk of postoperative death in BC patients. During the univariate Cox analysis of OS, the following variables were statistically significant in 175 patients: clinical T stage (HR = 2.790; *P* < 0.001), pathological T stage (HR = 1.764; *P* = 0.017), LNM (HR = 3.529; *P* < 0.001), PF (HR = 2.372; *P* = 0.001), NLR (HR = 2.513; *P* = 0.001), PLR (HR = 2.458; *P* < 0.001), LMR (HR = 2.117; *P* = 0.002), CRP (HR = 2.639; *P* < 0.001), and albumin (HR = 2.153; *P* = 0.002; Table 1). However, in the subgroup of 135 BC patients who had undergone RC, PLR (HR = 1.400; *P* = 0.169) was not significant (Table 2).

**Table 2:** Univariate and multivariate analyses of characteristics associated with OS in 136 BC undergoing RC patients

Characteristics	Univariate			Multivariate		
	Hazard ratio	95% CI	*P* value	Hazard ratio	95% CI	*P* value
**Age, years**						
≥60 vs <60	1.039	0.646–1.671	0.874			
**Gender**						
Male vs Female	1.540	0.962–2.465	0.072			
**Clinical T stage**						
>T2 vs ≤T2	2.018	1.235–3.299	**0.005**	1.825	1.108–3.007	**0.018**
**Pathological grade**						
G2–G3 vs G1	2.518	1.526–4.156	**<0.001**			
**Histological subtype**						
UBC vs Non-UBC	1.309	0.669–2.562	0.431			
**LNM**						
Yes vs No	3.350	1.929–5.820	**<0.001**	2.409	1.354–4.286	**0.003**
**PF**						
≥3.39 vs <3.39 g/L	2.591	1.459–4.602	**0.001**	3.759	1.360–10.390	**0.011**
**NLR**						
≥3.05 vs <3.05	2.353	1.414–3.925	**0.001**	4.337	1.426–13.191	**0.010**
**LMR**						
<3.06 vs ≥3.06	2.537	1.525–4.219	**<0.001**			
**PLR**						
≥138 vs <138	1.400	0.867–2.262	0.169			
**CRP**						
≥5.0 vs <5.0 mg/L	3.139	1.863–5.289	**<0.001**			
**Serum albumin**						
≥3.5 vs <3.5 g/dL	3.089	1.797–5.311	**<0.001**			
**PF + NLR**						
PF high + NLR high vs PF high or NLR high vs PF low + NLR low	4.896	1.902–12.937	**0.001**	0.264	0.077–0.906	**0.034**

*P* values that achieved statistical significance (*P* < 0.05) are indicated in boldface. Abbreviations: CRP = C-reactive protein; LMR = lymphocyte–monocyte ratio; LNM = lymph node metastasis; NLR = neutrophil–lymphocyte ratio; PF = plasma fibrinogen; PLR = platelet–lymphocyte ratio; UBC = urothelial bladder carcinoma.

### 3.3 Multivariate analysis of all prognostic parameters

Univariate values with *P* values less than 0.05 and prognostic factors were included in the multivariate analysis (Table 1). The NLR (HR = 4.419; *P* = 0.004), clinical T stage (HR = 2.320; *P* = 0.002), LNM (HR = 1.869; *P* = 0.035), and PF + NLR (HR = 0.294; *P* = 0.041) were independently associated with OS in all the patients. Additionally, the NLR (HR = 4.337; *P* = 0.010), clinical T stage (HR = 1.825; *P* = 0.018), PF (HR = 3.759; *P* = 0.011), PF + NLR (HR = 1.955; *P* = 0.003), and LNM (HR = 2.409; *P* = 0.003) were identified as independent prognostic factors of OS in those who had received RC treatment.

**Figure 1:**
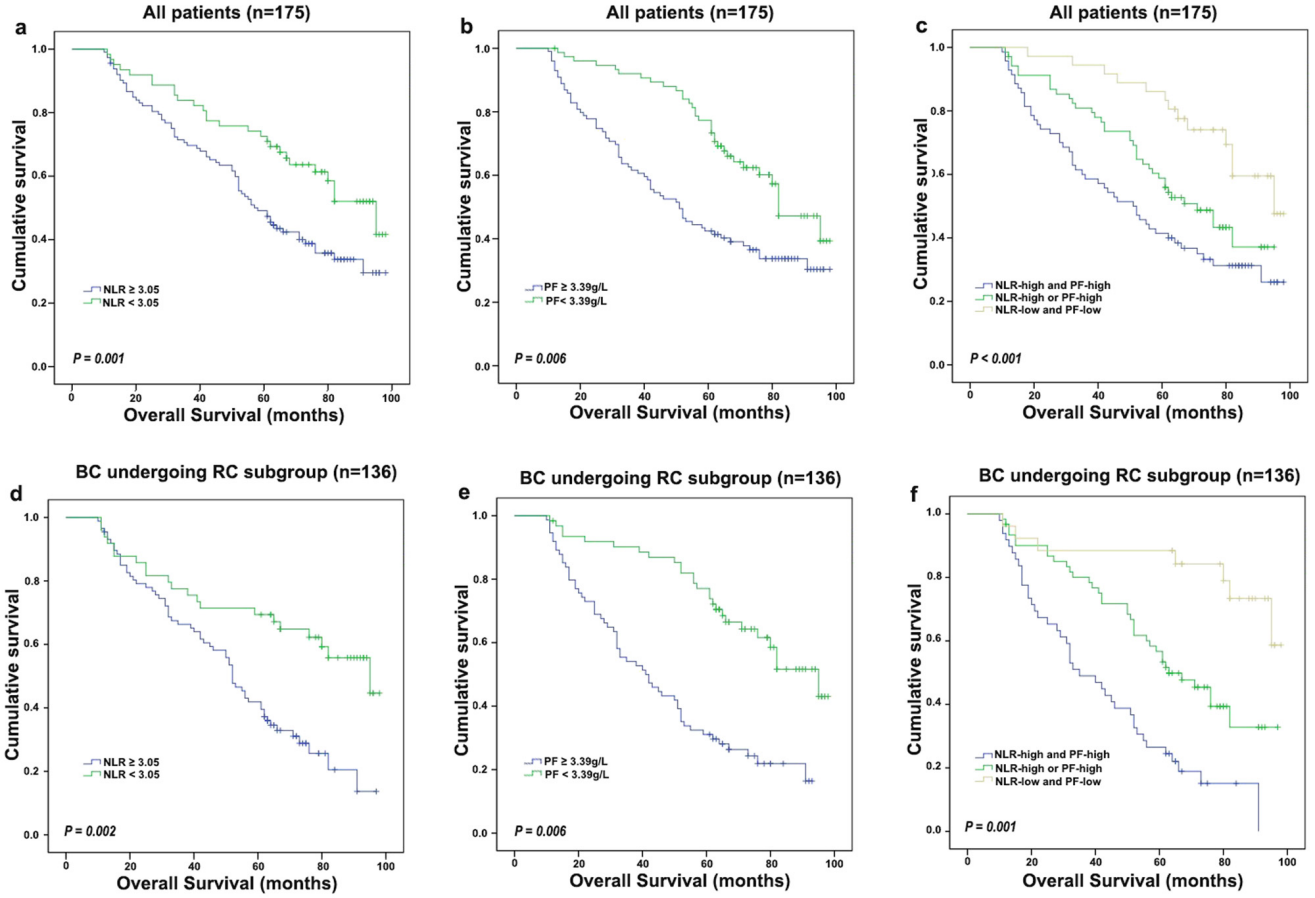
Prognosis significance of NLR and PF in all BC and BC following RC subgroup patients. The 5-year OS rate was calculated by the Kaplan–Meier method and analysed by the log-rank test. (a and d) OS based on NLR in all patients and RC subgroup. (b and e) OS based on PF in all patients and RC subgroup. (c and f) OS of combined NLR- and PF-based categorization in all patients and RC subgroup.

Kaplan–Meier analyses stratified by PF (Figure 1a and d) and NLR (Figure 1b and e) values showed that the elevation in these inflammation-based factors resulted in worse outcomes in all BC patients and those who had undergone RC. Dichotomization by the PF level demonstrated outcome differences among all the patients (5-year OS of 59.6% [PF high] vs 83.3% [PF low]), compared with dichotomization by the NLR value (5-year OS of 60.7% [NLR high] vs 82.5% [NLR low]). These results were similar to the subgroup of BC patients who had undergone RC, with estimated cumulative 5-year OS rates of 52.2% (PF high) and 78.3% (PF low) vs 50.0% (NLR high) and 76.8% (NLR low).

### 3.4 Associations of PF and NLR with clinicopathological factors in 136 BC patients following RC

The values of the clinicopathological variable were compared with the preoperative PF and NLR levels. LNM (*P* = 0.011) and an advanced clinical T stage (*P* = 0.015) were significantly correlated with a high PF level (≥3.39 g/L) in BC patients following RC (all *P*s < 0.05; Table 3). LNM (*P* < 0.001) and an advanced clinical T stage (*P* = 0.034) were significantly correlated with a high NLR value (≥3.05) in BC patients following RC (Table 3). Additionally, a significant positive correlation was found between a high PF level and a high NLR before surgery (*P* = 0.001; Table 3).

**Table 3:** Clinicopathological features of 136 BC undergoing RC patients stratified by PF and NLR

Characteristic	Total *n* = 136 (%)	PF			NLR		
		<3.39 g/L	≥3.39 g/L	*P* value	<3.05	≥3.05	*P* value
**Age (years),** ***n*** **(%)**							
<60	59	20	39	0.987	31	46	0.804
≥60	77	26	51		25	34	
**Gender,** ***n*** **(%)**							
Male	101	33	68	0.630	45	56	0.189
Female	35	13	22		11	24	
**Clinical T stage,** ***n*** **(%)**							
T2	63	28	35	**0.015**	32	31	**0.034**
T3	43	12	31		16	27	
T4	30	6	24		8	22	
**Pathological grade,** ***n*** **(%)**							
G1	67	27	40	0.116	33	34	0.059
G2	40	14	26		15	25	
G3	29	5	24		8	21	
**Histological subtype,** ***n*** **(%)**							
UBC	113	37	76	0.555	45	68	0.477
Non-UBC	23	9	14		11	12	
**LNM,** ***n*** **(%)**							
No	65	29	36	**0.011**	27	38	**<0.001**
Yes	71	17	54		18	53	
**NLR**							
≥3.05	80	18	62	**0.001**			
<3.05	56	28	28				

*P* values that achieved statistical significance (*P* < 0.05) are indicated in boldface. Abbreviations: LNM = lymph node metastasis; NLR **=** neutrophil–lymphocyte ratio; PF = plasma fibrinogen; UBC = urothelial bladder carcinoma.

### 3.5 Prognostic significance of the combination of PF and NLR

The results suggest a significant positive association between PF and NLR in 136 BC who had undergone RC (*P* = 0.001). The combination of NLR and PF can improve the stratification of BC patients. Therefore, we divided the BC patients into PF high and NLR high, NLR high or PF high, and NLR low and PF low, representing high-risk, intermediate-risk, and low-risk groups, respectively (Figure 1c and f). The 5-year OS rates of 175 BC patients in the high-, medium- and low-risk groups were 59.5, 62.3, and 97.4%, respectively. Similarly, the 5-year OS rates in 136 BC patients following RC were 50.0, 54.3, and 96.4%, respectively. In the multivariate model (Tables 1 and 2), both the high- and medium-risk groups showed lower OS rates than the low-risk group.

## 4 Discussion

BC is a heterogeneous urological neoplasm with different recurrence and progression rates [1]. Improved understanding of the molecular biology of BC has evolved how localized and advanced diseases are diagnosed and treated. The surgical treatment transurethral resection of bladder tumor and intravesical Bacillus Calmette-Guérin are applied for intermediate- and high-risk non-MIBC; the therapeutic options for muscle-invasive and advanced disease have expanded to neoadjuvant treatment [2,26]. However, controversy persists regarding which neoadjuvant treatment should be applied to certain patients, particularly those with intermediate- to high-risk muscle-invasive and advanced disease [27]. Thus, several predictors are required to provide additional support to determine which treatment groups should be applied in a specific high-risk group [28]. Recently, several inflammation-based biomarkers (e.g., albumin, CRP, NLR and PLR) have been associated with treatment outcomes in primary operable tumours [29,30]. Most studies have demonstrated the key role of neutrophils and fibrinogen in systemic inflammation. Increased numbers of neutrophils and fibrinogen indicate the presence of high-risk solid tumours [31,32].

Our data indicated the following: (1) high NLR and PF levels predict a lower survival rate of BC patients; (2) high NLR and PF levels are associated with a more aggressive clinical stage and LNM status in BC patients; (3) PF is a superior prognostic factor compared with the LMR, PLR, CRP, and albumin values in 136 BC patients who had undergone RC; (4) combining NLR and PF levels may improve the precision of survival outcome prediction in BC patients following RC.

The biological mechanism of PF explains its prognostic significance in BC. Many *in vitro* studies have verified that fibrinogen promotes carcinoma cell proliferation, angiogenesis, invasion, epithelial-to-mesenchymal transition (EMT), and haematogenous dissemination; therefore, it plays an important role in tumour development [33,34,35]. Other studies have found that fibrinogen binds to secreted growth factors, such as inhibition of apoptosis and members of the platelet-derived growth factor family, and induces tumour cell adhesion, vascular endothelial growth factor, metastasis, and fibroblast growth factor (FGF) families [36]. Furthermore, studies using cell line models have shown that fibrinogen may promote carcinoma cell motility by inducing the EMT via the p-AKT/p-mTOR pathway [37]. Additionally, *in vitro* studies reported that tumour cells can produce endogenous fibrinogen and the combination of FGF-2 and fibrinogen can stimulate the proliferation of endothelial cells, leading to enhanced angiogenesis [38,39].

A high NLR level is closely correlated with aggressive clinical features and is a significant risk factor affecting survival in BC patients. Neutrophils reflect a state of host inflammation, which can contribute to carcinoma onset [40]. They are involved in different stages of the tumour process, such as tumorigenesis, growth, proliferation, or metastasis [41,42]. Interestingly, the receptor tyrosine kinase MET is induced by tumour-derived tumour necrosis factor-α or other inflammatory stimuli in human neutrophils [43]. Finally, neutrophils also promote metastasis spreading by suppressing natural killer function and enhancing the extravasation of carcinoma cells [44,45]. Therefore, the prognostic value of plasma neutrophils as an independent factor or as a part of the NLR in carcinomas is evident because they promote neutrophil responses and/or lymphocyte suppression, leading to a high NLR, a suppressed anti-tumour immune response, and enhanced metastasis [46,47].

Inflammation, as a hallmark of carcinoma, affects all stages of tumorigenesis. Inflammasomes are a large complex of NOD-like receptors called NLRs, which have been identified as vital regulators in inflammation-related carcinogenesis, angiogenesis, metastasis, oxidative stress, cancer cell transformation, and chemoresistance [48,49]. The activity of NLRs is directly regulated by some miRNAs [50,51]. This suggests that the questing or synthesis of valuable chemotherapeutic drugs targeting these miRNAs could be a promising strategy for the treatment of BC.

Combining the PF level and NLR resulted in the lowest 5-year OS for patients in the PF high and NLR high groups. This finding further supports that high NLR and PF are related to the inflammatory response in BC patients, leading to an adverse outcome and further confirming that NLR correlates positively with PF. Additionally, this finding emphasizes that combining NLR and PF can better predict the prognosis in BC patients who have undergone RC than NLR or PF alone.

Our study has limitations. The first limitation is that our study is a retrospective analysis, and inherent choice bias cannot be eliminated. Additionally, our study evaluated a Chinese population; thus, the results cannot be generalized to other ethnic groups. More adequately designed prospective studies are required to further assess the BC patients.

In conclusion, the present study is the first to demonstrate that the PF level is an independent prognostic marker for predicting OS in BC patients who undergo RC. Additionally, the combination of PF and NLR can improve the prognostic accuracy and be used as a selection criterion for stratified treatment of risk factors in patients with BC.

## Appendix

**Table S1:** Clinicopathological features of 175 BC patients stratified by PF and NLR

Characteristic	Total *n* = 175 (%)	PF			NLR		
		<3.39g/L	≥3.39g/L	*P*-value	<3.05	≥3.05	*P*-value
**Age (years,** ***n*** **(%))**							
<60	74	28	46	0.875	28	46	0.750
≥60	101	35	66		35	66	
**Gender,** ***n*** **(%)**							
Male	124	54	70	0.160	56	68	0.089
Female	51	12	39		7	44	
**Clinical T stage,** ***n*** **(%)**							
Ta-1	35	13	22	0.005	15	20	0.082
T2	60	28	32		25	35	
T3	46	15	31		14	32	
T4	34	10	24		9	25	
**Pathological grade,** ***n*** **(%)**							
G1	70	30	40	0.318	33	37	0.737
G2	48	18	30		15	33	
G3	57	18	39		15	42	
**Histological subtype,** ***n*** **(%)**							
UBC	147	55	92	0.835	53	94	0.973
Non-UBC	28	11	17		10	18	
**LNM,** ***n*** **(%)**							
No	104	29	36	0.011	51	53	<0.001
Yes	71	17	54		12	59	
**NLR**							
≥3.05	112	28	84	<0.001			
<3.05	63	38	25				

*P*-values that achieved statistical significance (*p* < 0.05) are indicated in bold. Abbreviations: LNM = lymph node metastasis; NLR **=** neutrophil-lymphocyte ratio; PF = plasma fibrinogen; UBC = urothelial bladder carcinoma.
